# Anti-myeloma Effects of Icariin Are Mediated Through the Attenuation of JAK/STAT3-Dependent Signaling Cascade

**DOI:** 10.3389/fphar.2018.00531

**Published:** 2018-05-30

**Authors:** Young Yun Jung, Jong Hyun Lee, Dongwoo Nam, Acharan S. Narula, Ojas A. Namjoshi, Bruce E. Blough, Jae-Young Um, Gautam Sethi, Kwang Seok Ahn

**Affiliations:** ^1^College of Korean Medicine, Kyung Hee University, Seoul, South Korea; ^2^Narula Research, Chapel Hill, NC, United States; ^3^Center for Drug Discovery, RTI International, Research Triangle Park, Durham, NC, United States; ^4^Department for Management of Science and Technology Development, Ton Duc Thang University, Ho Chi Minh City, Vietnam; ^5^Faculty of Pharmacy, Ton Duc Thang University, Ho Chi Minh City, Vietnam; ^6^Department of Pharmacology, Yong Loo Lin School of Medicine, National University of Singapore, Singapore, Singapore

**Keywords:** icariin, STAT3, apoptosis, multiple myeloma, JAKs

## Abstract

Because of the essential role of signal transducer and activator of transcription 3 (STAT3) in proliferation, anti-apoptosis, and chemoresistance of multiple myeloma (MM), we investigated whether icariin, a prenylated flavonol glycoside, inhibits both constitutive and inducible STAT3 activation in human myeloma cell lines. We noted that icariin could block constitutive STAT3 phosphorylation as well as its nuclear translocation and DNA binding ability in U266 cells. Icariin also suppressed IL-6-induced STAT3 activation through the inhibition of upstream kinases (Janus activated kinase-1 and -2, and c-Src). We found that icariin downregulated the protein expression of STAT3 downstream target gene products such as Bcl-2, Bcl-xl, survivin, IAP-1/2, COX-2, VEGF, and matrix metallopeptidase 9 (MMP-9) in a concentration-dependent manner. Moreover, this flavonoid also exhibited the capacity to significantly induce apoptosis and suppress proliferation of MM cells. Interestingly, this agent also significantly potentiated the apoptotic effects of bortezomib through the suppression of STAT3 activation in MM cells. Altogether, our data indicates that the potential application of icariin as a STAT3 blocker in myeloma therapy.

## Introduction

Epimedium (family Berberidaceae), commonly called horny goat weed in the West that is known as Yin Yang Huo in Chinese medicine, is commonly used as a tonic, aphrodisiac, anti-rheumatic and anti-cancer agent in traditional herbal remedies in China, other parts of Asia (Liu et al., 2006; Li C. et al., 2015; Tan et al., 2016). The herb contains a highly potent active ingredient named icariin, which is also the source of many of the potential health benefits (Lee et al., 1995; Lin et al., 2004). In a number of recent studies, icariin has shown potent anti-tumor activity in very broad classes of cancer cell types such as gastric (Wang et al., 2010), liver (Li S. et al., 2010; Li et al., 2014), gallbladder (Zhang et al., 2013), colon (Zhang et al., 2014), breast (Ma et al., 2014), ovarian (Li J. et al., 2015), and esophageal cancer cells (Fan et al., 2016; Gu et al., 2017). These results suggest that icariin is a promising lead compound with high efficiency in cancer prevention and treatment as also reported with various other agents derived from natural sources (Shanmugam et al., 2011; Tang et al., 2014; Bishayee and Sethi, 2016).

Multiple myeloma, also known as plasma cell myeloma, is a cancer of plasma cells characterized by bone marrow infiltration by malignant plasma cells, which produce monoclonal immunoglobulin (Ig) fragments (Kastrinakis et al., 2000; Kannaiyan et al., 2011, 2012; Sikka et al., 2014; Baek et al., 2017b). Despite significant advances in scientific understanding and clinical management of MM, it remains a nearly uniformly fatal disease, with the currently available therapeutic strategies producing a mean 5-year survival rate of 49% based on MM. (Statistics obtained from www.cancer.net).

Conventionally, the therapeutic regimens implemented for the treatment of MM using alkylating agents (melphalan), and corticosteroids, can extend patient survival by an average of 3-4 years (Kannaiyan et al., 2012; Suzuki, 2013; Sikka et al., 2014). Although the combination of high-dose chemotherapy and hematopoietic stem cell transplantation has shown a significant improvement in lifespan in MM patient, not all patients are eligible for this regimen and the recurrence commonly prevails with complex drug resistant phenotypes (Hultcrantz et al., 2012; Kannaiyan et al., 2012; Kumar et al., 2012; Sikka et al., 2014). As a result, treatment and the development of strategies for MM should be re-considered, because targeted therapies based on enhanced understanding of signaling networks have imparted clinical benefit (Kannaiyan et al., 2012; Yap et al., 2013; Sikka et al., 2014).

There is currently strong evidence to suggest that aberrant activation of signal transducer and activator of transcription 3 (STAT3) represents a key step in the neoplastic process in human cancers through the induction of anti-apoptosis, cell proliferation, angiogenesis, invasion, and metastasis (Siveen et al., 2014; Chai et al., 2016; Shanmugam et al., 2016; Wong et al., 2017). The analyses of primary tumor cells from patients with MM and established MM cells have consistently revealed that STAT3 is constitutively active in approximately 40–60% of MM tumors (Catlett-Falcone et al., 1999; Quintanilla-Martinez et al., 2003; Bharti et al., 2004; Kannaiyan et al., 2012; Sikka et al., 2014). It has been previously found that a reduction in the expression of various negative regulators of IL6/JAK/STAT pathway by epigenetic silencing can also sensitize myeloma cells to IL-6-regulated proliferation and survival (Galm et al., 2003; Kannaiyan et al., 2012; Sikka et al., 2014). Interestingly, overexpression of SOCS abrogated IL-6 induced proliferation in MM cells, thereby suggesting another possible way to abrogate IL-6 induced downstream STAT3 signaling cascade (Bommert et al., 2006; Yamamoto et al., 2006; Kannaiyan et al., 2012; Kolosenko et al., 2014; Sikka et al., 2014).

Moreover, various experimental studies have demonstrated that STAT3 inhibition has been generally well-tolerated in normal cells (Subramaniam et al., 2013; Siveen et al., 2014). Therefore, STAT3 inhibition has become an attractive target for cancer therapy, because it has a strong potential to offer broader clinical impact. Here, we investigated whether icariin could inhibit the aberrant activation of STAT3 signaling pathway in human myeloma cell lines (U266 and MM.1S). Therefore, our data clearly shows that icariin repressed both constitutive and IL-6-induced STAT3 activation, inhibited JAK-1/2 and c-Src activation, and down-regulated various gene products that are regulated by STAT3, thus leading to suppression of proliferation and induction of apoptosis. Also, icariin was found to synergistically enhance both the cytotoxic and pro-apoptotic effects of bortezomib in MM cells.

## Materials and Methods

### Reagents

**FIGURE 1:**
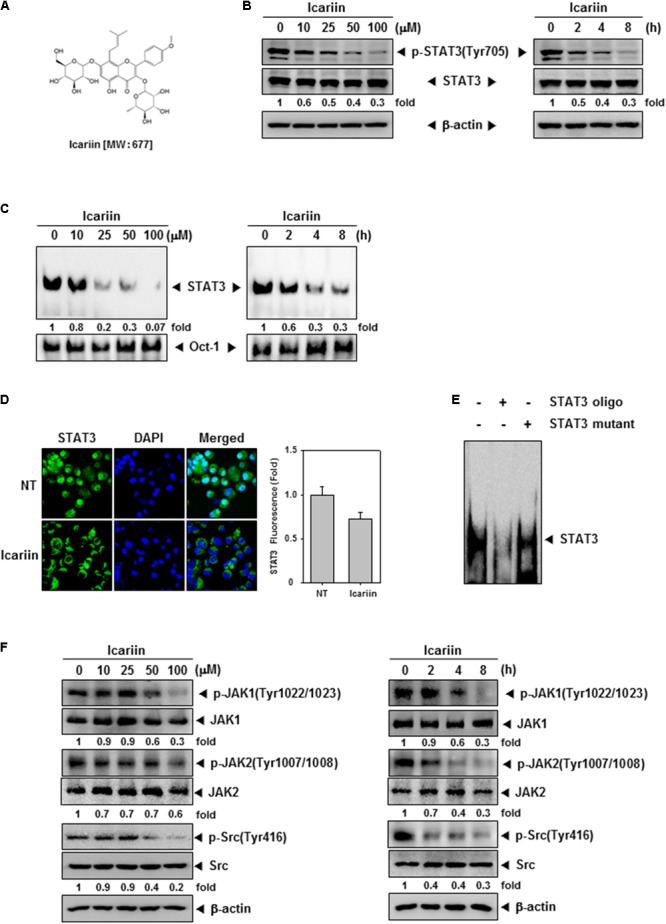
Icariin downregulates the constitutive STAT3, JAK1/2, and Src in U266 cells. **(A)** Chemical structure of icariin. **(B)** U266 cells (1 × 10^6^ cells/well) were treated with the indicated concentrations of icariin for 8 h and different time periods with 100 μM. Then the same amounts of whole cell lysates were prepared and compared to assess inhibition for phospho-STAT3(Tyr705), and STAT3 by western blot analysis. **(C)** U266 cells (1 × 10^6^ cells/well) were treated with various indicated concentrations and different time periods. Thereafter nuclear extracts were studied for STAT3 inhibition levels by EMSA. **(D)** U266 cells (2 × 10^4^ cells/well) were treated with 100 μM of icariin for 6 h, and analyzed distribution of STAT3 into the nucleus by immunocytochemistry. Datas were quantified on graph (*right panel*). **(E)** Specificity of STAT3 was confirmed by competition assay in U266 cells. Nuclear extract of U266 cells were binding with STAT3 consensus oligonucleotide (*second lane*) or mutant oligonucleotide (*third lane*). **(F)** U266 cells (1 × 10^6^ cells/well) were treated with various indicated concentrations of icariin and different time periods. Then the expression of p-JAK1(Tyr1022/1023), JAK1, p-JAK2(Tyr1007/1008), JAK2, p-Src(Tyr416), and Src were analyzed by western blotting. The results shown are representative of three independent experiments.

Icariin (**Figure 1A**) was purchased from Sigma-Aldrich (St. Louis, MO). Icariin stock solution (100 mM) was prepared in dimethyl sulfoxide, storage at -20°C and finally diluted in cell culture medium to use. RPMI 1640, fetal bovine serum (FBS), and penicillin-streptomycin mixture and LightShift^®^ Chemiluminescent EMSA kit were purchased from Thermo Fisher Scientific Inc. (Waltham, MA). 3-(4,5-dimethylthiazol-2-yl)-2,5-diphenyltetrazolium bromide (MTT), propidium iodide (PI), Tris base, glycine, NaCl, sodium dodecylsulfate (SDS), and bovine serum albumin (BSA) were purchased from Sigma-Aldrich (St. Louis, MO). 5′-biotinylated STAT3 was obtained from Bioneer Corporation (Daejeon, Korea). Alexa Fluor^®^ 488 donkey anti-rabbit IgG (H+L) antibody was obtained from Life Technologies (Grand Island, NY). Anti-phospho-STAT3(Tyr705), anti-phospho-JAK1(Tyr1022/1023), anti-JAK1, anti-phospho-JAK2(Tyr1007/1008), anti-JAK2, anti-phospho-Src(Tyr416), and anti-cleaved-caspase-3 antibodies were purchased from Cell Signaling Technology (Beverly, MA). Anti-STAT3, anti-Src, anti-Bcl-2, anti-Bcl-xL, anti-Survivin, anti-IAP-1, anti-IAP-2, anti-COX-2, anti-VEGF, anti-MMP-9 (matrix metalloproteinase-9), anti-caspase-3, anti-PARP, anti-p21 and anti-β-actin antibodies were purchased from Santa Cruz Biotechnology (Santa Cruz, CA). QIAprep^®^ Spin Miniprep Kit was obtained from QIAGEN. TUNEL (terminal transferase mediated dUTP-fluorescein nick end labeling) assay kit was from Roche Diagnostics GmbH (Mannheim, Germany). Annexin V staining kits (ApoScan) were purchased from BioBud (Seoul, Korea).

### Cell Lines

Human MM cell U266 and MM.1S were obtained from American Type Culture Collection (Manassas, VA). U266 and MM.1S cells were cultured in RPMI 1640 medium containing 10% FBS, 1% penicillin and streptomycin.

### Western Blotting

After treating with icariin for various indicated concentrations and time points, cells were harvested and lysed with 1 × cell lysis buffer. Then protein concentration in the whole cell lysates was measured by Bradford reagent (Bio-Rad, Hercules, CA, United States). Equal amounts of lysates were separated by their protein size on sodium dodecyl-polyacrylamide gel electrophoresis (SDS–PAGE) and transferred to nitrocellulose membrane, blocked with 5 or 3% skim milk in 1 × TBST (1 × TBS with 0.1% Tween 20) for 2 h at room temperature. After blocking, membranes were incubated at 4°C for overnight with specific primary antibodies: anti-phospho-STAT3(Tyr705), anti-STAT3, anti-phospho-JAK1(Tyr1022/1023), anti-JAK1, anti-phospho-JAK2(Tyr1007/1008), anti-JAK2, anti-phospho-Src(Tyr416), anti-Src, anti-Bcl-2, anti-Bcl-xL, anti-Survivin, anti-IAP-1, anti-IAP-2, anti-COX-2, anti-VEGF, anti-MMP-9 (matrix metalloproteinase-9), anti-caspase-3, anti-PARP and anti-cleaved-caspase-3. Finally, membranes were washed three times using 1 × TBST, and incubated with horseradish peroxidase (HRP) conjugated anti-rabbit IgG antibodies and anti-mouse IgG antibodies at room temperature for 2 h. Then proteins were detected by enhanced chemiluminescence (ECL) (EZ-Western Lumi Femto, DOZEN). After detection, stripping the membrane for 1 h and we demonstrated that equal amounts of whole proteins were loaded by probing membranes with anti-β-actin antibodies.

### EMSA for STAT3-DNA Binding

Electrophoretic mobility shift assay (EMSA) was performed to analyze STAT3-DNA binding. Cells were treated for the indicated time periods and concentrations with icariin and nuclear extract was prepared using 10 × binding buffer, poly(di-dc), NP-40, and probe. 5′-biotinylated STAT3 oligonucleotide (5′-GATCCTTCTGGGAATTCCTAGATC-3′ and 5′-GATCTAGGAATTCCCAGAAGGATC-3′; BIONEER, Daejeon, Korea) in complex with nuclear protein and Oct-1 (5′-TTCTAGTGATTTGCATTCGACA-3′ and 5′-TGTCGAATGCAAATCACTAGAA-3′; BIONEER, Daejeon, Korea) was used for loading control. Protein-oligonucleotide complex was loaded on polyacrylamide gel and transferred to nylon membrane, then cross-linked by 540 nm UV. Finally, protein expression was detected by using LightShift^®^ Chemiluminescent EMSA kit (Waltham, MA).

### EMSA for STAT3-Competition Assay

Electrophoretic mobility shift assay (EMSA) was performed to analyze STAT3 specificity. Nuclear extract was prepared using 30 × unlabeled STAT3 consensus oligonucleotide (GATCCTTCTCGGGAATTCCTAGATC-3′; BIONEER, Daejeon, Korea) or mutant STAT3 oligonucleotide (GATCCTTCTGGGCCGTCCTAGATC-3′ BIONEER, Daejeon, Korea) incubation for 20 min in room temperature. After oligonucleotide binding reaction, added the 10 × binding buffer, poly(di-dc), NP-40, and probe then incubation for 20 min in room temperature. Samples were loaded on 6% polyacrylamide gel and transferred to nylon membrane, then cross-linked by 540 nm UV.

### Immunocytochemistry for STAT3 Localization

U266 cells were treated with 100 μM icariin for 8 h and centrifuged in a Shandon CytoSpin III Cytocentrifuge. Cells were fixed with 4% paraformaldehyde (PFA) at room temperature for 20 min and washed by 1 × PBS, permeabilised by 0.2% triton X-100. Then blocked using 5% BSA for 1 h and incubated with anti-STAT3 (1:100; Santa Cruz, CA) for overnight at 4°C. Next, cells were washed with 1 × PBS and incubated with Alexa Fluor^®^ 488 donkey anti-rabbit IgG (H+L) antibody at room temperature for 1 h. Then, stained with DAPI (1 μg/ml) for 3 min at room temperature and mounted in Fluorescent Mounting Medium (Golden Bridge International Labs, Mukilteo, WA, United States). Finally, the fluorescence signal was detected by using an Olympus FluoView FV1000 confocal microscope (Tokyo, Japan)μ.

### Reverse Transcription Polymerase Chain Reaction (RT-PCR) for RNA Analysis

U266 cells were treated with icariin (0, 10, 25, 50, and 100 μM) for 8 h then washed with 1 × PBS. Cells were suspended in Trizol, and RNA purified using chloroform and isopropanol. Then RNA was Reverse transcribed into cDNA using superscript reverse transcriptase and Taq polymerase by reverse transcription polymerase chain reaction (RT-PCR) (TAKARA, Tokyo, Japan). Glyceraldehyde-3-phosphate dehydrogenase (GAPDH) was used as control. For quantitative PCR analyses, Bcl-2, Bcl-xl, and Survivin were polymerized using the following primers: Bcl-2, 5′-TTGTGGCCTTCTTTGAGTTCGGTG-3′ and 5′-TACAGTTCCACAAAGGCATCCCAG-3′. Bcl-xl, 5′-TACCAGCCTGACCAATATGGC-3′ and 5′-TGGGTTCAAGTGATTCTCCTG-3′. Survivin, 5′-GATGACGACCCCATGCAAA-3′ and 5′-TTTCTCCGCAGTTTCCTCAAA-3′. RT-PCR was performed with Bcl-c at 94°C for 15 s, 58°C for 30 s, 72°C for 1 min with 28 cycles and extension at 72°C for 5 min. Bcl-xl reaction was performed at 94°C for 30 s, 57°C for 30 s, 72°C for 1 min with 30 cycles and extension at 72°C for 7 min. Survivin reaction was performed at 94°C for 30 s, 55°C for 30 s, 72°C for 30°C with 30 cycles and extension at 72°C for 7 min. Finally, PCR products were separated on 1% agarose gel and stained with Loading Star (Dynebio, Seongnam, Korea). Stained bands were detected by UV light.

### Transfection Assay

MM.1S cells (2 × 10^6^ cells/well) were transfected by electroporation 1150 v, 30 ms with STAT3-luciferase DNA (300 ng) and STAT3 dominant-negative DNA (300 ng) in 10% FBS-supplemented RPMI 1640 medium. After 48 h incubation for transfection, we resuspended the cells with icariin (0, 10, 25, 50, and 100 μM) in culture medium 1 ml for 8 h and cells were treated with IL-6 (10 ng/ml) for 10 min. Cells were harvested and lysed with 1× lysis buffer (Reporter Lysis 5× Buffer) for overnight incubation in -80°C. To each sample (20 μl of protein in water) 40 μl luciferase assay reagent was added and luminescence was measured, in a luminometer.

### Cell Transfection and siRNA Knockdown

To inhibit STAT3 expression in U266 cells by RNA interference, U266 cells (2 × 10^6^ cells/well) were transfected with 50 nM STAT3 siRNA (sc-29493; Santa Cruz Biotechnology) or 100 nM scrambled siRNA (SN-1002; BIONEER, Daejeon, Korea) using NEON Transfection system (Invitrogen). Then cells were incubated in 10% FBS-supplemented RPMI 1640 medium for 48 h.

### Cell Cycle Analysis

Cell cycle analysis was performed to examine the effects of icariin on cell cycle progression. U266 cells (1 × 10^6^ cells/well) were treated with 100 μM icariin for 24 h, harvested and washed with 1 × PBS and incubated with 1 mg/ml RNase A in 1 × PBS at 37°C for 1 h. Then cells were washed, and stained with 25 μg/ml propidium iodide in 1 × PBS for at least 30 min at room temperature. Stained cells were analyzed by FACScan Calibur flowcytometry (BD Biosciences, Becton-Dickinson, Franklin Lakes, NJ, United States) with Cell Quest 3.0 software.

### Annexin V Assay

To confirm that icariin can induce early apoptosis in U266 cells, we performed the Annexin V assay. After U266 cells (1 × 10^6^ cells/well) were treated with 100 μM icariin for 24 h, cells were harvested and washed. Then stained with FITC tagged Annexin V antibody in 1 × PBS protected from light at room temperature for 15 min. Then stained with 25 μg/ml propidium iodide and analyzed with Cell Quest 3.0 software.

### TUNEL Assay

To evaluate synergistic effects between icariin and bortezomib to induce apoptosis, we treated U266 cells for 24 h with icariin (10 μM) and bortezomib (1 nM). Treated cells were fixed with 4% paraformaldehyde for 30 min, washing in PBS and resuspendin PBS overnight. After fixation cells were washed with PBS and treated with 0.2% triton X-100 for 10 min. Finally, cells were washed again with PBS, stained with TUNEL enzyme and TUNEL label for 1 h at 37°C analyzed by FACScan Calibur flowcytometry (BD Biosciences, Becton-Dickinson, Franklin Lakes, NJ, United States) with Cell Quest 3.0 software.

### MTT Assay

Cell viability was measured using an MTT assay. Both U266 cells and PBMC cells (1 × 10^4^ cells/well) were treated with icariin (0, 10, 25, 50, and 100 μM) for 24 h. After treatment, 2 mg/ml MTT solution 30 μl was added on each well for 2 h and 100 μl MTT lysis buffer was added for overnight incubation. Finally, we measured absorbance using automated spectrophotometric plate reader at 570 nm. Cell viability was normalized as relative percentages in comparison with untreated controls.

### Live and Dead Assay

U266 cells were treated with 100 μM icariin for 24 h and centrifuged by Shandon CytoSpin III Cytocentrifuge. We used Live and Dead assay (Invitrogen, Carlsbad, CA, United States). Cells were stained with 5 μM Calcein AM and 5 μM Ethd-1(Ethidium homodimer-1) at 37°C for 30 min. Live cells have intracellular esterase activity that converts Calcein AM into intensely fluorescent calcein producing green color. On the other hand, dead cells have damaged cellular membrane then Ethd-1 can invade into cell, combine with nucleic acid and produce bright red fluorescence. Stained cells were detected by Olympus FluoView FV1000 confocal microscope (Tokyo, Japan).

### Combination Therapy With Bortezomib and Icariin

To confirm the combination effect between icariin (0, 10, 25, and 50 μM) and Bortezomib (0, 1, 2.5, and 5 nM), U266 cells (1 × 10^4^ cells/well) were seeded on 96 well plate and treated with each concentration mixture for 24 h. First, we using MTT assay to optimize treatment conditions. Next, cells were evaluated by CalcuSyn (BIOSOFT, Ferguson, MO, United States) software. Input each data to calculate a combination index (CI) and select moderate combination rate. Using these data, synergy and also antagonism can be evaluated: CI < 1, CI = 1 and CI > 1, respectively.

### Statistical Analysis

All numerical values are represented as the mean ± SE. Statistical significance of the data compared with the untreated control was determined using the Mann-Whitney *U*-test. Significance was set at *p* < 0.05.

## Results

### Icariin Suppresses Constitutive Phosphorylation of STAT3 in Human Multiple Myeloma Cells

First, we tested whether icariin suppressed constitutive STAT3 activation. U266 cells were treated with icariin at various concentrations (0, 10, 25, 50, and 100 μM) for 8 h, and 100 μM icariin for different time intervals (0, 2, 4, and 8 h). We used whole cell extract, probed with p-STAT3(Tyr705) and STAT3 antibodies. As shown in **Figure 1B**, icariin suppression of p-STAT3(Tyr705) was concentration-dependent (**Figure 1B**, left) and time-dependent (**Figure 1B**, right), but there was no effect on STAT3 basal level. We found that icariin exhibited maximum inhibitory effect at around 100 μM concentration after treatment for 8 h.

### Icariin Inhibits STAT3 DNA Binding Activity and Nuclear Translocation in MM Cells

Because dimerized STAT3 can translocate into nucleus and induce transcription of target genes, we tested whether icariin can inhibit STAT3 binding to DNA. EMSA analysis showed that in nuclear extract from U266 STAT3-DNA binding inhibition by icariin was concentration and time-dependent (**Figure 1C**). Results show that icariin had suppressive effects on STAT3-DNA binding ability. Activated STAT3 dimers can translocate into nucleus and induce transcription of specific genes, we visualized that icariin can inhibit nuclear translocation of STAT3. As shown in **Figure 1D**, icariin-treated cells showed reduced STAT3 translocation into nuclei compared with NT cells. These results show that icariin inhibits STAT3 translocation into nuclei. Additionally to test the specificity of STAT3 ability to bind to the DNA, competition assay was performed, 5 μg of nuclear extracts were incubated with 30× unlabeled consensus STAT3 oligonucleotide or mutant STAT3 oligonucleotide. The protein-DNA complex was effectively blocked by 30× unlabeled consensus STAT3 on STAT3-binding site (**Figure 1E**, lane 2), however 30× unlabeled mutant STAT3 oligonucleotide did not prevent the protein-DNA complex (**Figure 1E**, lane 3).

### Icariin Represses Constitutive JAK1, JAK2, and Src Activation

STAT3 is known to be activated by Janus family (JAK) and Src (Chai et al., 2016; Wong et al., 2017). To determine if icariin also downregulates upstream signaling kinases involved with STAT3 signaling pathway U266 cells were treated with various concentrations of icariin for 8 h. U266 cells were treated for different time intervals with 100 μM icariin. As shown in **Figure 1F**, p-JAK1, p-JAK2, and p-Src were downregulated by icariin in both concentration (left) and time-dependent (right) manners. These results show that icariin also downregulates activation of signaling kinases upstream of STAT3.

### Icariin Inhibits Inducible Activation of STAT3 and Upstream Kinases in MM.1S Cells

**FIGURE 2:**
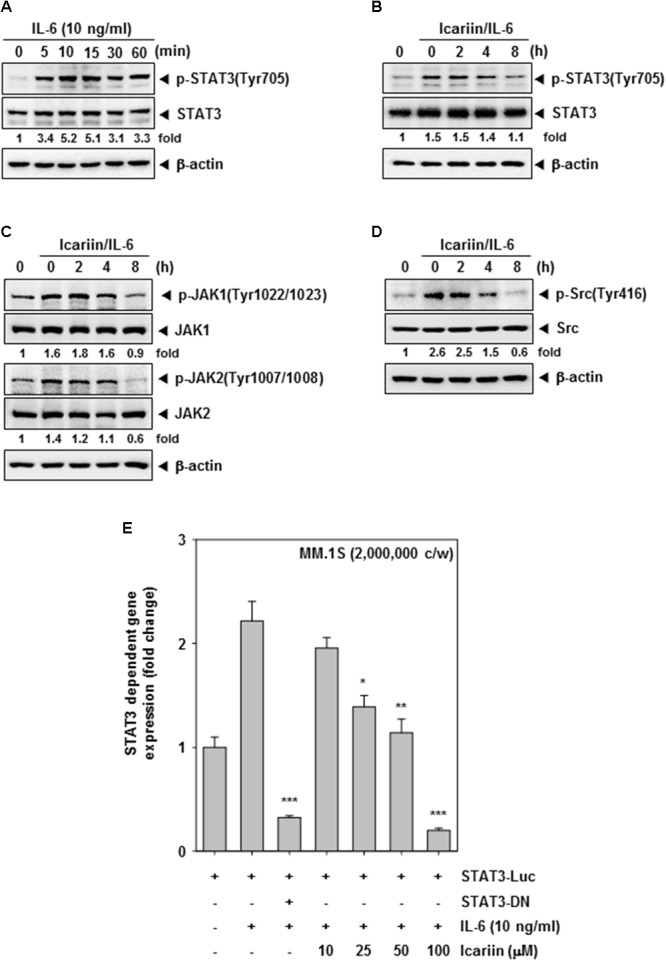
Icariin can inhibit inducible STAT3 in MM.1S cells. **(A)** MM.1S cells (1 × 10^6^ cells/well) were treated with IL-6 (10 ng/ml) for different time periods. Then whole cell lysates were compared for p-STAT3(Tyr705) and STAT3 expression by western blot analysis to determine the optimal periods of p-STAT3 signaling. **(B–D)** MM.1S cells (1 × 10^6^ cells/well) were treated with 100 μM icariin for different time periods and then stimulated with IL-6 (10 ng/ml) for 10 min. Equal amounts of whole cell lysates were prepared and expression of p-STAT3(Tyr705), STAT3, p-JAK(Tyr1022/1023), JAK1, p-JAK(Tyr1007/1008), JAK2, p-Src(Tyr416), and Src were analyzed by western blotting. **(E)** STAT3 promoter luciferase assay in MM.1S cells (2 × 10^6^ cells/well) transfected for 48 h, and were treated with icariin (0, 10, 25, 50, 100 μM) for 8 h. Then stimulated with IL-6 (10 ng/ml) for 10 min. The results shown are representative of three independent experiments.

Next, we tested whether icariin can inhibit inducible STAT3 signaling in MM.1S cells. Because IL-6 induces STAT3 activation, we treated with IL-6 (10 ng/ml) for various intervals (0, 5, 10, 15, 30, and 60 min) to select the optimal time point. As shown in **Figure 2A**, there was minimal signaling initially, but an increase p-STAT3 signaling was detected at 10 min after IL-6 exposure. MM.1S cells (1 × 10^6^ cells/well) were incubated with 100 μM icariin for different time intervals (0, 2, 4, and 8 h) then stimulated with IL-6 (10 ng/ml) for 10 min. Whole cell lysates were prepared and analyzed by Western blotting. IL-6-induced p-STAT3(Tyr705) was clearly suppressed by icariin but there was no effect on STAT3 basal level (**Figure 2B**). We next investigated whether icariin has suppressive effects on STAT3-related upstream signaling kinases. First we pretreated MM.1S cells (1 × 10^6^ cells/well) with 100 μM icariin for 8 h and IL-6 (10 ng/ml) were treated for 10 min. Inducible phospho-JAK1(Tyr1022/1023) and phospho-JAK2(Tyr1002/1008) signals were clearly reduced by icariin with no effects on total JAK1 and JAK2 protein levels (**Figure 2C**). Also, inducible phospho-Src (Tyr416) expression was suppressed by icariin but the level of total Src protein showed no change (**Figure 2D**).

### Icariin Inhibits IL-6–Induced STAT3-Dependent Reporter Gene Expression

We transiently transfected MM.1S cells (2 × 10^6^ cells/well) with a STAT3-luciferase construct and a STAT3 dominant-negative construct, then cells were pretreated with icariin (0, 10, 25, 50, and 100 μM) for 8 h and then IL-6 (10 ng/ml) for 10 min. As shown in **Figure 2E**, STAT3-mediated luciferase gene expression significantly increased after IL-6 treatment but dominant-negative STAT3 blocked this increase, indicating specificity. When the cells were pre-incubated with icariin, IL-6-induced STAT3 activity was suppressed in a dose-dependent manner.

### Icariin Suppresses Anti-apoptosis, Proliferation, Angiogenesis, and Metastasis Related Proteins

**FIGURE 3:**
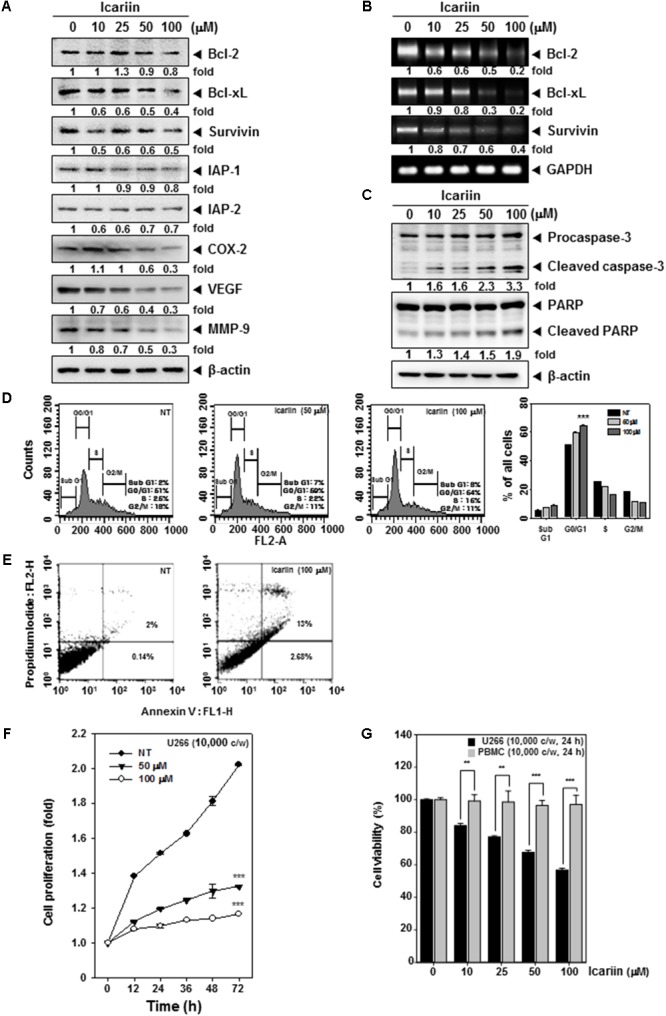
Icariin induces apoptosis in U266 cells. **(A)** U266 cells (1 × 10^6^ cells/well) were treated with various indicated concentrations for 24 h. Then 10 μg proteins of those whole cell extracts was loaded on 15% SDS–PAGE gel followed by western blot analysis against Bcl-2, Bcl-xl, Survivin, IAP-1, IAP-2, COX-2, VEGF, and MMP-9. **(B)** U266 cells (1 × 10^6^ cells/well) were treated with various indicated concentrations for 8 h. Total RNA was extracted and equal amounts were prepared to probe for Bcl-2, Bcl-xl, and Survivin by RT-PCR. **(C)** U266 cells (1 × 10^6^ cells/well) were treated with various indicated concentrations for 24 h. Equal amounts of whole cell lysates were prepared and performed to detect caspase-3 and PARP by western blotting. **(D)** After Icariin treatment (0, 50, 100 μM for 24 h), cells were washed with PBS and digested with RNase A for 1 h, then stained with propidium iodide and analyzed cell for cycle division using flow cytometry. **(E)** After U266 cells (1 × 10^6^ cells/well) were incubated with 100 μM icariin for 24 h, stained with Annexin V-FITC for 15 min and add PI then were analyzed by flow cytometry. **(F)** U266 cells (1 × 10^4^ cells/well) were incubated with icariin (0, 50, 100 μM) for different indicated time periods. After incubation, difference in degree of cell proliferation was analyzed by MTT assay. **(G)** U266 cells (1 × 10^4^ cells/well) and PBMC cells (1 × 10^4^ cells/well) were incubated with 100 μM icariin for 24 h. The results shown are representative of three independent experiments.

Various proteins such as Bcl-2, Bcl-xl, Survivin, IAP-1, IAP-2, COX-2, VEGF, and MMP-9 have been shown to have activities affecting anti-apoptosis, proliferation, angiogenesis, and metastasis (Chai et al., 2016). We examined whether icariin has inhibitory effects on these proteins, U266 cells (1 × 10^6^ cells/well) were incubated with various concentrations (0, 10, 25, 50, and 100 μM) for 24 h. As shown in **Figure 3A**, icariin had suppressive effects on anti-apoptosis related proteins such as Bcl-2, Bcl-xl, Survivin, IAP-1, and IAP-2. Also icariin suppressed cell proliferation, angiogenesis, and metastasis related proteins such as COX-2, VEGF, and MMP-9. Icariin also suppressed Bcl-2, Bcl-xl, and Survivin at the mRNA levels (**Figure 3B**).

### Icariin Activates Caspase-3 and Causes PARP Cleavage

Whether suppression of constitutively active STAT3 in U266 cells by icariin leads to apoptosis was investigated. U266 cells (1 × 10^6^ cells/well) were treated with various concentrations (0, 10, 25, 50, 100 μM) for 24 h. We found that caspase-3 activation was induced by icariin in a concentration-dependent manner. Interestingly, the activation of caspase-3 also induced the PARP cleavage. These results show that icariin induces caspase-3-dependent apoptotic pathway in U266 cells (**Figure 3C**).

### Icariin Induces Cell Cycle Arrest and Promotes Apoptosis in U266 Cells

We were also interested in examining the effects of icariin on cell cycle progression in U266 cells. After icariin treatment (0, 50, and 100 μM) for 24 h, cells were stained with PI and analyzed by FACScan Calibur flow cytometry (BD Biosciences, Becton-Dickinson, Franklin Lakes, NJ, United States) with Cell Quest 3.0 software. As shown in **Figure 3D**, icariin-induced an increased accumulation of cell population in G0/G1 phases and a corresponding decrease of cells in S and G2/M phases on U266 cells. To evaluate the anti-tumor effects of icariin, we also examined the apoptosis-inducing effects of icariin by using the Annexin V assay and observed by flow cytometric analysis. U266 cells were treated with 100 μM icariin for 24 h. As shown in **Figure 3E**, icariin increased early apoptosis in U266 cells. It reached up to 13% at 100 μM icariin compared with non-treated cells (2%).

### Icariin Suppresses the Viability of MM Cells Without Affecting the Normal Cells

We next examined whether icariin can suppress cell viability in U266 cells by MTT assay. U266 cells (1 × 10^4^ cells/well) were treated with icariin (0, 50, and 100 μM) for 72 h and measured at every 12 h intervals. After icariin treatment, 30 μl MTT solution (2 mg/ml) were given for 2 h and 100 μl MTT lysis buffer were given for overnight incubation. As shown in **Figure 3F**, viability of U266 cells was suppressed by both 50 μM and 100 μM icariin compared with non-treated cells (^∗∗∗^*p* < 0.001 was considered statistically significant). To test whether icariin was also cytotoxic to normal cells, we used peripheral blood mononuclear cells (PBMC). PBMC (1 × 10^4^ cells/well) and U266 cells (1 × 10^4^ cells/well) were seeded and treated with various concentrations (0, 10, 25, 50, and 100 μM) for 24 h. After icariin treatment, 30 μl MTT solution (2 mg/ml) were added for 2 h and 100 μl MTT lysis buffer added for overnight incubation. Next we analyzed cell viability by automated spectrophotometric plate reader at 570 nm. Results show that the viability was clearly reduced by icariin in U266 cells, but PBMC maintained their viability (**Figure 3G**).

### Icariin Enhances the Cytotoxic Effect of Bortezomib

**FIGURE 4:**
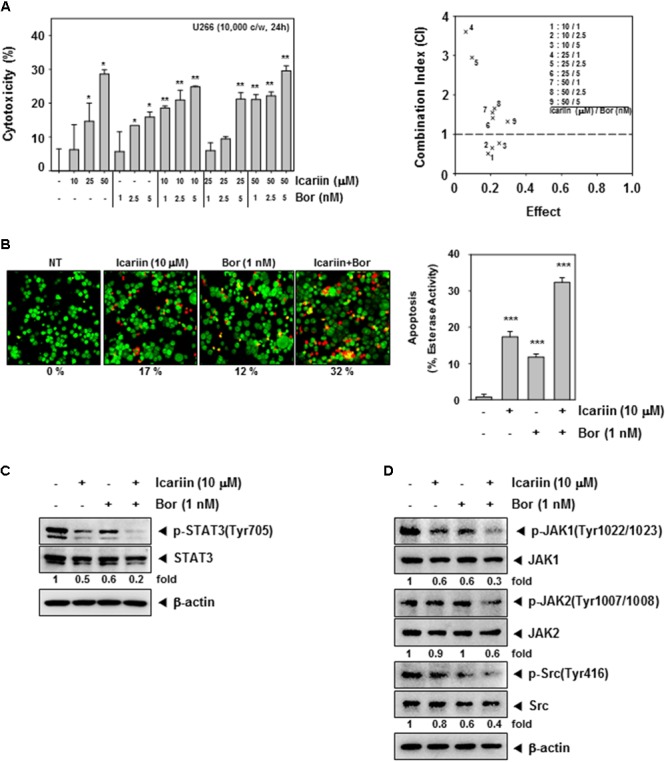
Icariin enhance Bortezomib in U266 cells. **(A)** To confirm the synergic effect on cytotoxicity of icariin and bortezomib (Bor), we used the MTT assay. U266 cells (1 × 10^4^ cells/well) were incubated with icariin (0, 10, 25, and 50 μM) and bortezomib (0, 1, 2.5, and 5 nM) for 24 h. The average of CI values for various combinations shows that icariin increase cytotoxicity of bortezomib. The best combination ratio is 10 μM icariin and 1 nM bortezomib. **(B)** Live and Dead assay was performed to confirm the synergic effects on U266 cells (1 × 10^6^ cells/well) with 10 μM icariin and 1 nM bortezomib for 24 h. Live cells were stained in green and dead cells were stained in red. The graph (*right)* shows the rate of dead cells by quantification. **(C,D)** U266 cells (1 × 10^6^ cells/well) were treated with 10 μM icariin and 1 nM bortezomib for 24 h. Then equal amounts of whole cell lysates were prepared and expression of p-STAT3(Tyr705), STAT3, p-JAK1(Tyr1022/1023), JAK1, p-JAK2(Tyr1007/1008), JAK2, p-Src(Tyr416), and Src were analyzed by western blotting. The results shown are representative of three independent experiments.

First, we examined cell viability using an MTT assay to confirm the synergic cytotoxicity between icariin and bortezomib. U266 cells (1 × 10^4^ cells/well) were seeded then treated with various combinations of icariin (0, 10, 25, and 50 μM) and bortezomib (0, 1, 2.5, and 5 nM) for 24 h. According to Calcusyn software (BIOSOFT, Ferguson, MO), optimal ratio of combination was 10 μM icariin with 1 nM bortezomib (**Figure 4A**). To determine the synergistic effects on apoptosis, we first examined cell viability using the Live and Dead assay. U266 cells were treated with both icariin (10 μM) and bortezomib (1 nM) for 24 h. Then stained with 5 μM Calcein AM and 5 μM Ethd-1(Ethidium homodimer-1) at 37°C for 30 min. Finally, live cells stained with green color and dead cells stained with red color were detected by Olympus FluoView FV1000 confocal microscope (Tokyo, Japan). As shown in **Figure 4B**, combination of icariin and bortezomib treated cells showed more induction of apoptosis compared with cells treated with icariin or bortezomib separately.

### Combination of Icariin and Bortezomib Substantially Inhibits Constitutive STAT3 Activation and Its Upstream Kinases

We examined whether the optimal concentrations had synergistic effect on constitutive STAT3 activation and its upstream signaling kinases. U266 cells (1 × 10^6^ cells/well) were treated with icariin (10 μM) and bortezomib (1 nM) then analyzed by Western blotting. Results show that compared with icariin and bortezomib alone, the combination had synergistic suppression effects on p-STAT3(Tyr705), p-JAK1(Tyr1022/1023), p-JAK2(Tyr1002/1008), and p-Src(Tyr416) activation but there has no reduction seen in STAT3, JAK1, JAK2, and Src proteins (**Figures 4C,D**).

### Combination of Icariin and Bortezomib Augments G0/G1 Phase Cell Cycle Arrest and Cellular Apoptosis

**FIGURE 5:**
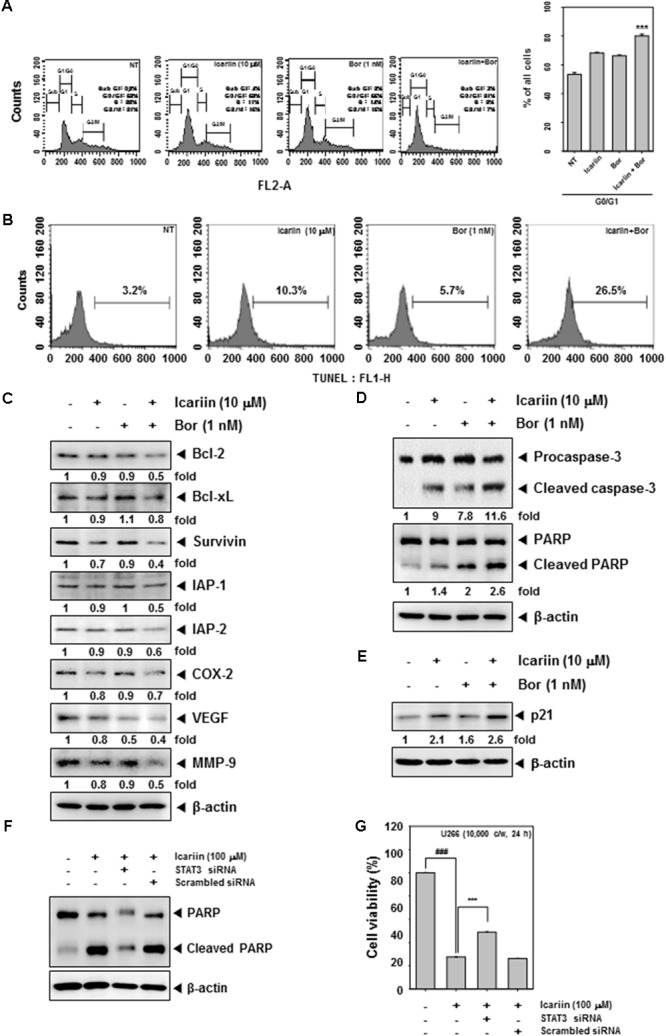
Icariin and Bortezomib induce apoptosis by caspase-3 and PARP in U266 cells. **(A)** To confirm the synergistic effect between icariin and bortezomib on cell cycle, U266 cells (1 × 10^6^ cells/well) were incubated with icariin (10 μM) and bortezomib (1 nM) for 24h then treated with RNase A for 1h. After staining with propidium iodide, cells were analyzed by flow cytometry. **(B)** U266 cells were treated with icariin and bortezomib for 24 h. Cells were fixed and stained with TUNEL assay reagent, then analyzed with a flow cytometer. **(C,D)** We confirm the synergistic effect for induced apoptosis by western blot analysis. U266 cells (1 × 10^6^ cells/well) were treated with 10 μM icariin and 1 nM bortezomib for 24 h. Same amounts of whole cell lysates were prepared and probed using Bcl-2, Bcl-xl, Survivin, IAP-1, IAP-2, COX-2, VEGF, MMP-9, caspase-3, and PARP antibodies then analyzed by Western blotting. **(E)** To confirm the anti-cancer effect of icariin and bortezomib, U266 cells (1 × 10^6^ cells/well) were incubated with icariin (10 μM) and bortezomib (1 nM) for 24h then proteins were resolved on SDS–PAGE and probed against p21 antibody. **(F)** U266 cells were transfected with STAT3 siRNA or scramble siRNA for 48 h, then 100 μM of icariin were treated for 24 h. The Whole cell lysates were prepared and 15 μg proteins were resolved on SDS–PAGE and probed against PARP antibody. b-actin was used as internal controls. **(G)** U266 cells were transfected with STAT3 siRNA or scramble siRNA for 48 h, then 100 μM of icariin were treated for 24 h. Then cell viability was analyzed by MTT assay. The results shown are representative of three independent experiments.

We determined whether icariin enhances bortezomib suppression of cell cycle progression in U266 cells. Cells were co-incubated with icariin and bortezomib for 24 h. 1 mg/ml RNase A was treated for 1 h in 37°C and then with stained 25 mg/ml propidium iodide at room temperature. Cells were analyzed by FACScan Calibur flow cytometry (BD Biosciences, Becton-Dickinson, Franklin Lakes, NJ, United States) with Cell Quest 3.0 software. As shown in **Figure 5A**, G0/G1 phase was increased to 81% by the combination treatment compared with non-treated cells 52%, icariin (68%), and bortezomib (66%) alone. U266 cells were treated with icariin (10 μM) and bortezomib (1 nM) for 24 h. Cells were fixed with 4% paraformaldehyde and permeabilize with 0.2% Triton x-100 then stained with TUNEL enzyme and label. Finally, we analyzed by FACScan Calibur flow cytometry (BD Biosciences, Becton-Dickinson, Franklin Lakes, NJ, United States) with Cell Quest 3.0 software. TUNEL-positive cells were observed about 10.3% (icariin alone) and 5.7% (bortezomib alone). In cells treated with the combination of icariin and bortezomib tunne-positive cells were increased up to 26.5% (**Figure 5B**). Icariin and bortezomib have synergistic effects for apoptosis compared with icariin and bortezomib alone.

### Icariin Exerts Synergistic Effect With Bortezomib in Suppressing the Expression of Various Oncogenic Proteins

Next, we determined whether icariin has synergistic effects with bortezomib on Bcl-2, Bcl-xl, Survivin, IAP-1, IAP-2, COX-2, VEGF, and MMP-9. U266 cells (1 × 10^6^ cells/well) were treated with icariin and bortezomib for 24 h and detected by Western blot analysis. Anti-apoptosis proteins (Bcl-2, Bcl-xl, Survivin, IAP-1, and IAP-2) were more downregulated with combination treatment, and also proliferation proteins (COX-2) and angiogenesis proteins (VEGF, MMP-9) were more suppressed by the combination compared with icariin and bortezomib alone (**Figure 5C**). In addition, caspase-3, PARP cleavage and p21 expression was found to be further increased upon the co-treatment of icariin along with bortezomib rather than treatment with individual agents alone (**Figures 5D,E**). Overall, these results show that combined treatment with icariin along with bortezomib increased apoptosis in MM cells as compared with either agents alone.

### Inhibition of STAT3 by siRNA Reverses the Observed Pro-apoptotic Effects of Icariin

To provide a direct evidence that the functional effects observed in the presence of icarrin are due to inhibition of JAK/STAT pathway, STAT3 expression was blocked by using STAT3 siRNA and the effect on apoptosis was confirmed by performing western blot analysis against PARP and measurement of cell viability by MTT assay. As shown in **Figures 5F,G**, icariin-induced apoptosis was significantly abolished upon transfection with STAT3 siRNA as compared to the scrambled control.

## Discussion

Multiple myeloma is a malignant cancer of the plasma cells characterized by cytogenetic abnormalities and is a feature in patients with monoclonal gammopathy of uncertain significance (MGUS), the first stage that may progress to myeloma (Anderson, 2011a, b; Abdi et al., 2013). Almost all patients with myeloma have cytogenetically abnormal tumor cells and often do not respond to conventional chemotherapy (Anderson, 2011a; Kannaiyan et al., 2012; Sikka et al., 2014). Thus development of MM is complex and hetergogenous and the transformation is dependent of bone marrow microenvironment and subsequent additional mutations drive MM cells to the transformation of extramedullary MM (Agarwal and Ghobrial, 2013; Sikka et al., 2014). MM cells are more often found adhered to stromal cells that secrete IL-6 and the secreted cytokine acts in a paracrine fashion and drives MM cells to proliferate at a faster pace by activating the prosurvival JAK/STAT signaling pathway (Kannaiyan et al., 2011, 2012; Sikka et al., 2014; Chai et al., 2015).

Constitutively active STAT3 is often encountered in several types of cancer cells including MM and plays a pivotal role in cancer cell survival and proliferation (Li F. et al., 2010; Kannaiyan et al., 2012; Subramaniam et al., 2013; Sikka et al., 2014; Chai et al., 2016; Shanmugam et al., 2016). Therefore, suppression of constitutively active STAT3 in MM cells provides an opportunity to inhibit MM cell proliferation and survival. Several natural product compounds have been shown to inhibit the JAK/STAT signaling pathway in diverse cancer cells and preclinical models including MM cells (Li F. et al., 2010; Kannaiyan et al., 2011; Shanmugam et al., 2011; Yang et al., 2013; Lee et al., 2014; Siveen et al., 2014; Tang et al., 2014; Hsieh et al., 2015; Bishayee and Sethi, 2016; Baek et al., 2017a,b). Indeed, icariin has been shown to inhibit the growth of human esophageal carcinoma cells by inhibiting the PI3K/AKT and STAT3 signaling pathways (Gu et al., 2017). However, the anti-cancer effects of icariin in MM cells had not been extensively investigated, although icaritin a hydrolitic product of icariin has been reported to modulate IL-6/JAK2/STAT3 signaling cascade in MM (Zhu et al., 2015). Interestingly, icaritin not only inhibited tumor growth but also decreased serum IL-6 and IgE levels, without exhibiting any adverse effects such as body weight loss (Zhu et al., 2015). In another recent study, it was reported that icariin could reverse multidrug resistance in human osteosarcoma MG-63 doxorubicin-resistant (MG-63/DOX) cells through the blockade of STAT3 phosphorylation (Wang et al., 2018).

The aim of this study was to determine whether icariin could suppress the proliferation of MM cells and augment the cytotoxic effects of bortezomib by interfering with the STAT3 signaling pathway. We found that icariin inhibited U266 cell proliferation in a dose dependent manner and did not have any cytotoxic effect on PBMC cells. In addition, icariin selectively inhibited in a dose and time dependent manner the phosphorylation of tyrosine 705 on STAT3. Our results clearly indicate that inhibition of Y705 phosphorylation of STAT3 by icariin may be mediated by downregulation of phosphorylation of multiple upstream kinases such as JAK1/JAK2 and Src. Interestingly, it was also observed that icariin treatment induced the inhibition of Src activation as early as 2 h and this suppression of Src activation may act together with JAK kinases to abrogate STAT3 activation. IL-6 is a known inducer of STAT3 phosphorylation and roles of upstream kinases such as JAK1, JAK2, Src have been implicated in STAT3 transcriptional activation (Kannaiyan et al., 2012; Sikka et al., 2014; Chai et al., 2016). We also found that icariin inhibited constitutive and IL-6 inducible STAT3 activation with the abrogation of p-JAK1 and p-JAK2 and p-Src activation in MM cells. We further observed that icariin suppressed STAT3 nuclear translocation and IL-6-induced reporter activity of STAT3. This finding suggests that icariin could exert its effect on STAT3 activation through modulating multiple steps of STAT3 activation pathway.

**FIGURE 6:**
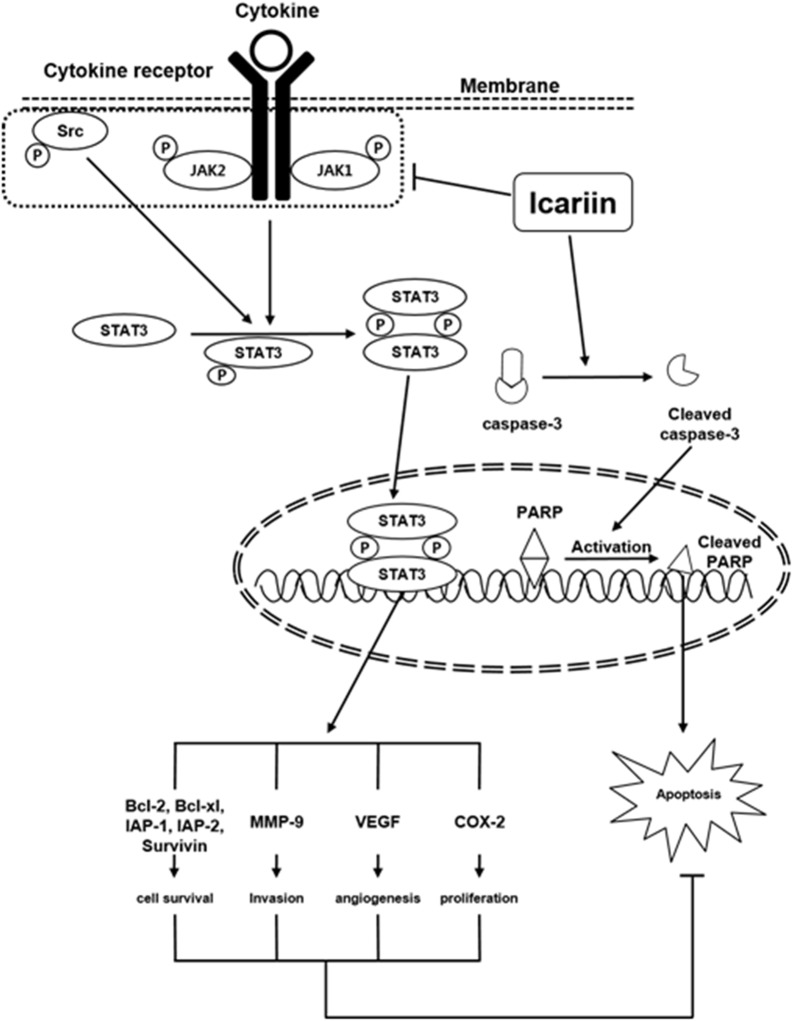
A schematic diagram showing the effects of icariin on STAT3 signaling pathways and apoptosis in MM cells.

Several lines of evidences suggests that the expression of STAT3 regulated genes such as survivin, Bcl-2, Bcl-xL confers resistance to apoptosis in human breast cancer cells (Gritsko et al., 2006) and it has been reported that these anti-apoptotic genes play an important role in the development of chemoresistance mechanisms (Tu et al., 1998; Kannaiyan et al., 2012; Sikka et al., 2014). Icariin also downregulated the expression of STAT3 regulated gene products such as Bcl-2, Bcl-xL, survivin, IAP-1, IAP-2, COX-2, VEGF and MMP-9. Icariin treatment induced significant accumulation of sub-G1 phase cells and induced caspase-mediated apoptosis. Food and Drug Administration (FDA) approved the drug, bortezomib, a proteasome inhibitor for the treatment of MM (Li F. et al., 2015; Scalzulli et al., 2018). We also found that icariin can potentiate the apoptotic effects of bortezomib in MM cells as evidenced by the increase in sub-G1 population of cells, which was associated with the suppression of anti-apoptotic proteins. Furthermore, we observed that icariin augments the apoptotic effects in the presence of bortezomib and that the antiproliferative/pro-apoptotic effects of icariin were predominantly mediated through inhibition of the STAT3 signaling pathway. Our results clearly show that icariin inhibits IL-6 signaling quite effectively. Our results indicate for the first time that icariin inhibits both inducible and constitutive STAT3 activation, which makes it a potentially effective suppressor of tumor cell survival, proliferation and angiogenesis. A schematic diagram of the effects of icariin on STAT3 signaling pathways and apoptosis in MM cells is presented in **Figure 6**. Further *in vivo* studies may provide important leads for using icariin as treatment of cancers carrying STAT3 activating mutations.

## Author Contributions

YJ, JL, and DN designed the project and performed the experiments. AN, ON, BB, J-YU, GS, and KA analyzed the data and compiled the manuscript.

## Conflict of Interest Statement

The authors declare that the research was conducted in the absence of any commercial or financial relationships that could be construed as a potential conflict of interest.
